# Metal Free Graphene Oxide (GO) Nanosheets and Pristine-Single Wall Carbon Nanotubes (p-SWCNTs) Biocompatibility Investigation: A Comparative Study in Different Human Cell Lines

**DOI:** 10.3390/ijms19051316

**Published:** 2018-04-28

**Authors:** Federica Valentini, Emanuela Mari, Alessandra Zicari, Andrea Calcaterra, Maurizio Talamo, Maria Giovanna Scioli, Augusto Orlandi, Stefania Mardente

**Affiliations:** 1 Department of Sciences and Chemical Technologies, University of Rome Tor Vergata, via della Ricerca Scientifica 1, 00133 Rome, Italy; 2 INUIT Foundation, University of Roma Tor Vergata, via dell’Archigginasio, 00133 Rome, Italy; andrea.calcaterra@uniroma2.it (A.C.); maurizio.talamo@uniroma2.it (M.T.); 3 Department of Experimental Medicine, University of Rome Sapienza, Viale Regina Elena 324, 00161 Rome, Italy; emanuela.mari@uniroma1.it (E.M.); alessandra.zicari@uniroma1.it (A.Z.); stefania.mardente@uniroma1.it (S.M.); 4 Department of Medicine, Pathological Anatomy, University of Rome Tor Vergata, Viale Oxford 81, 00133 Rome, Italy; scioli07@hotmail.it (M.G.S.); augusto.orlandi@uniroma2.it (A.O.)

**Keywords:** p-SWCNTs, graphene oxide, biocompatibility, cytotoxicity, LDH

## Abstract

The in vitro biocompatibility of Graphene Oxide (GO) nanosheets, which were obtained by the electrochemical exfoliation of graphite electrodes in an electrolytic bath containing salts, was compared with the pristine Single Wall Carbon Nanotubes (p-SWCNTs) under the same experimental conditions in different human cell lines. The cells were treated with different concentrations of GO and SWCNTs for up to 48 h. GO did not induce any significant morphological or functional modifications (demonstrating a high biocompatibility), while SWNCTs were toxic at any concentration used after a few hours of treatment. The cell viability or cytotoxicity were detected by the trypan blue assay and the *lactate dehydrogenase* LDH quantitative enzymatic test. The Confocal Laser Scanning Microscopy (CLSM) and transmission electron microscopy (TEM) analysis demonstrated the uptake and internalization of GO sheets into cells, which was localized mainly in the cytoplasm. Different results were observed in the same cell lines treated with p-SWCNTs. TEM and CLSM (Confocal Laser Scanning Microscopy) showed that the p-SWCNTs induced vacuolization in the cytoplasm, disruption of cellular architecture and damage to the nuclei. The most important result of this study is our finding of a higher GO biocompatibility compared to the p-SWCNTs in the same cell lines. This means that GO nanosheets, which are obtained by the electrochemical exfoliation of a graphite-based electrode (carried out in saline solutions or other physiological working media) could represent an eligible nanocarrier for drug delivery, gene transfection and molecular cell imaging tests.

## 1. Introduction

In recent years, scientific communities have devoted a considerable effort to evaluate the biocompatibility of nanomaterials with the aim of ensuring their safe use in the emerging fields of nanomedicine and nano-pharmacology [1,2]. Graphene (G) represents a promising nanomaterial for several biomedical applications due to its unique atomic structure that allows its functionalization, low cytotoxic profile in biosystems [3] and high surface area-to-volume ratio. This ensures a relevant loading capacity of the bioactive molecules, genes and miRNA for drug/miRNA delivery and gene transfection purposes [4,5]. Furthermore, the surface chemistry of graphene-based materials, which is strictly related to their functionalization degree and composition [6,7], can be specifically modulated to enhance exceptional optical and electrical properties in G that are useful for in vitro cell imaging and in vivo monitoring of these nanomaterials during experimentations [8,9,10]. For this purpose, G derivatives, such as Graphene Oxides (GO), Reduced Graphene (RG) and Graphene NanoComposites (GNCs), have been widely investigated in terms of pharmacokinetics, toxicity and cytocompatibility [11,12,13]. Currently, pristine Single Wall Carbon Nanotubes (p-SWCNTs) are widely studied for their effects on cells, animals and environment in addition to being evaluated for their biosafety [14,15,16]. Many studies on p-SWCNTs show that they exhibit higher in vitro and in vivo toxicity in biological systems compared with other G derivatives, because their fiber shape can cause cellular damages. Furthermore, the carbon nanotubes contain contaminants, such as heavy metals (employed as catalysts for their assembly), and their diameter represents a crucial structural aspect as it affects functionalization [17]. It has been previously reported that the biocompatibility of p-SWCNTs improved after binding with bio-polymers [18]. Although our previous work [19] demonstrated that functionalized (i.e., oxidized SWCNTs) nanotubes exhibit certain levels of toxicity in embryos, we compared the biocompatibility of p-SWCNTs with GO nanosheets in different human cell lines in this present study.

Regarding the GO biosafety, many recent studies have demonstrated its biocompatibility in cells or living biosystems [20,21,22]. These promising effects could be partially explained by the metal-free composition of GO and its geometrical structure, which are namely sheets and not nanotubes. Currently, the most representative studies focus on the application of GO in the Henrietta Lacks cells (HeLa) derived from cervical cancer tissue and Human Umbilical Vein Endothelial cells (HUVEC), which are both selected as the models of stable epithelial and endothelial cell lines, respectively. Furthermore, GO has been also applied in the Human Dermal Fibroblast (HDF) and NIH 3T3 (RRID:CVCL_0594) murine fibroblast cells [23]. Experimental evidence shows that GO exhibits dose-dependent toxicity in both human and mice [24,25] cells. This aspect is of crucial importance when nanomaterials are used for drug delivery applications, gene transfection and other biomedical purposes. In this regard, we are reporting on the cytotoxicity effects of GO and p-SWCNTs on different cell lines (the human endothelial-like immortalized cell line EaHy926 derived from fusion of HUVEC cells with the bronchial carcinoma cell line-A549, the human laryngeal epidermoid carcinoma HEp-2 cells, HUVEC and HeLa) in this present work by exploring the cell viability in the presence of GO and p-SWCNTs, especially in terms of exposure times and concentration of these nanomaterials. Cellular uptake, distribution and morphological changes induced by GO and p-SWCNTs into the cells were monitored by Confocal Laser Scanning Microscopy (CLSM).

## 2. Results

### 2.1. Nanomaterials Characterization

**Figure 1:**
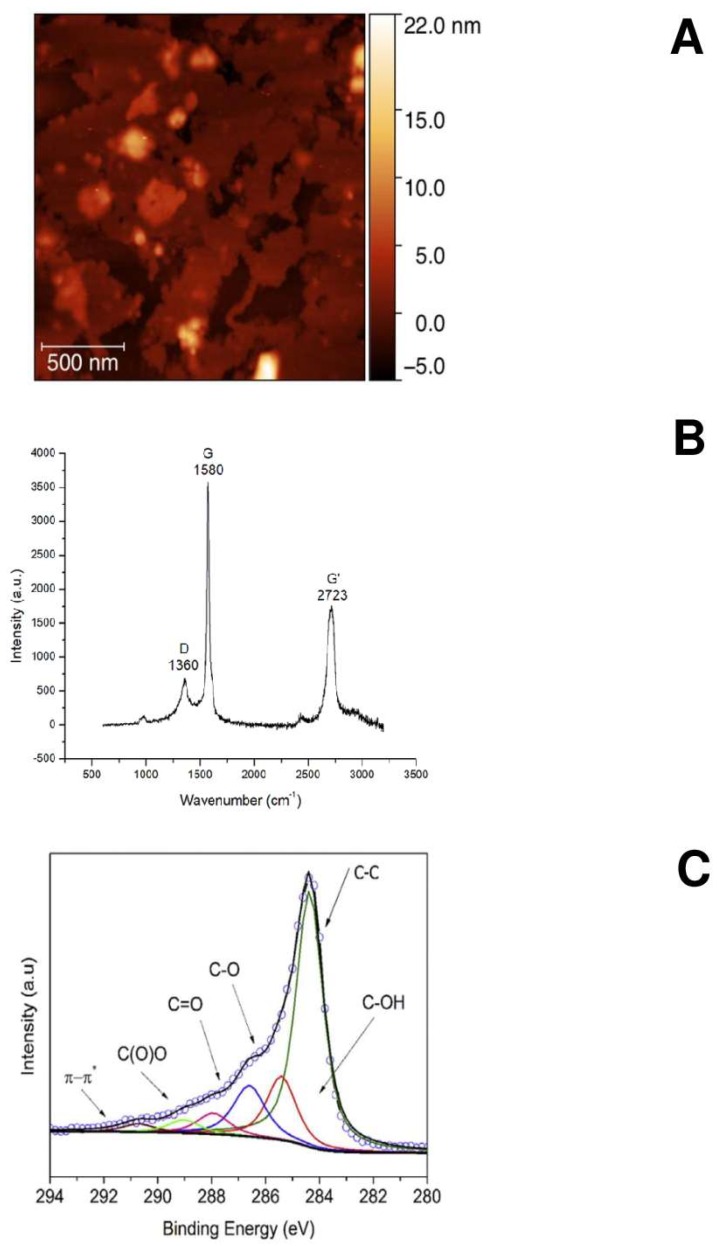
(**A**) AFM of GO nanosheets; (**B**) Raman spectrum of GO and (**C**) XPS with the C1s peaks.

Figure 1 shows the Atomic Force Microscopy (AFM), Raman spectrum and X-ray Photoelectron Spectroscopy (XPS) of GO nanosheets. A complete chemical–physical characterization of GO is summarized in Table 1 and Table 2. Figure 1A shows the AFM of GO organized in 2 layers. The Raman spectrum (Figure 1B) shows GO defects (D peak) in the 2 layers (G′). The defects shown in the Raman spectrum are better defined in Figure 1C and in Table 2, where the XPS reveals and quantifies carboxylic, carbonyl, ether and alcoholic groups. Furthermore, the thermal analysis and acidic sites determination assays revealed that the GO surfaces and sides have a low number of oxidized functional groups [26]. The thickness, surface area and number of layers were estimated by AFM analysis and are reported in Table 1. The elemental compositional analysis revealed the complete absence of metals, confirming the “metal-free” nature of these GO derivatives.

The chemical–physical properties of p-SWCNTs are reported in Table 3. The elemental analysis revealed the presence of Si, S, Fe, Ca, Cr and Co as the main impurities, which are used as catalysts for the growth of p-SWCNTs. Considering that the metallic elements could be responsible for cytotoxicity (and for this reason, a soft physical purification procedure has been applied in order to minimize the metal content in nanotubes), the concentration of metals in p-SWCNTs was quantified by using the ICP-OES (Inductively Coupled Plasma Optical Emission Spectrometry) analytical method. Among the possible contaminants, only cobalt was found at a final concentration of 30 μM. The oxygenated functionalities on walls, sides and surfaces in the p-SWCNTs could be related to the air annealing purification treatment. p-SWCNTs were further cut with various cycles of sonication (see Section 4) which leads to a reduction of approximately 30% in length.

### 2.2. Effects of GO and p-SWCNTs Treatments on Cell Lines

The cytotoxicity of p-SWCNTs and GO at 0.2 µg/mL and 2 µg/mL was measured by quantifying the release of lactate dehydrogenase (LDH) in the different cell lines and by the trypan blue exclusion test (shown in Table 4 and Table 5).

**Figure 2:**
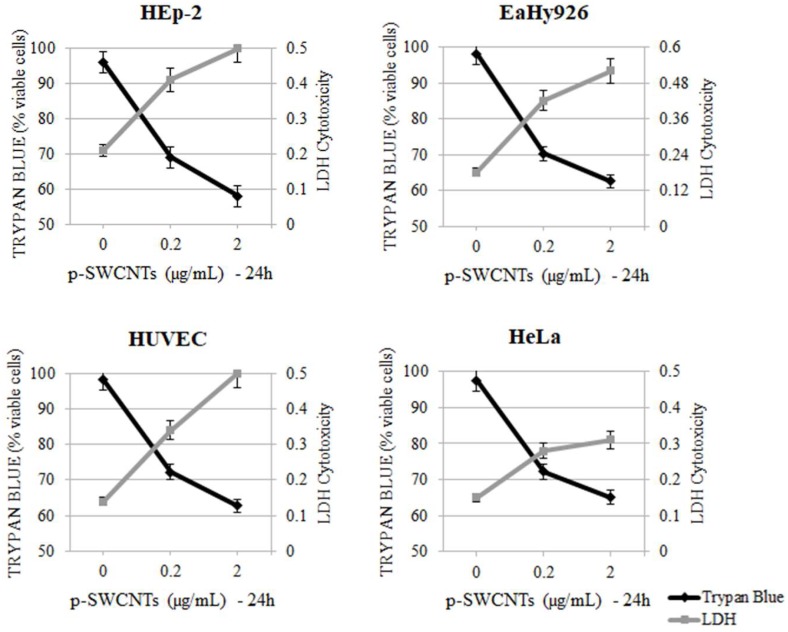
Effect of p-SWCNTs at different concentrations (0.2 and 2 µg/mL) for 24 h on HEp-2, EaHy926, HUVEC and HeLa cells. The viability detected with trypan blue exclusion test was correlated to LDH cytotoxicity. HEp-2 (2 µg/mL vs. control) *p* = 0.002 (LDH and Trypan blue); HUVEC (2 µg/mL vs. control) *p* = 0.001; EaHy926 (2 µg/mL vs. control) *p* = 0.002; HeLa (2 µg/mL vs. control) *p* = 0.003.

**Figure 3:**
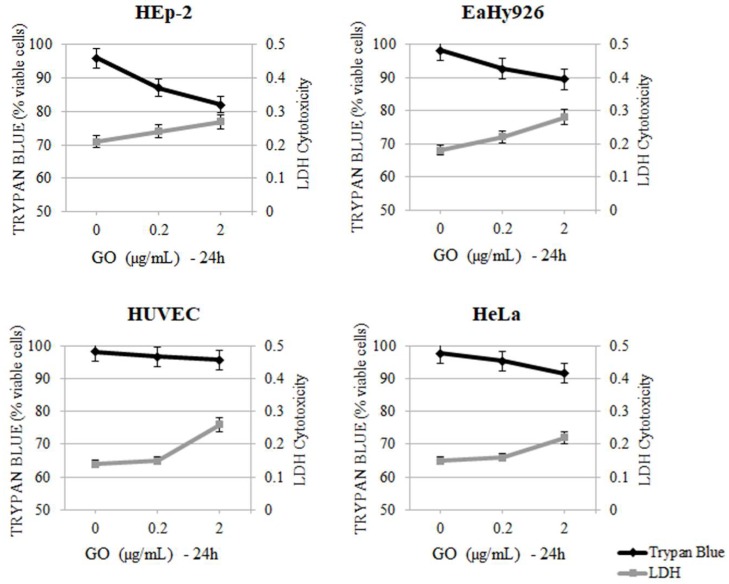
Effect of GO at different concentrations (0.2 and 2 µg/mL) for 24 h on HEp-2, EaHy926, HUVEC and HeLa cells. Viability detected with Trypan blue exclusion test was correlated to LDH (2 µg/mL GO vs. control HEp-2 *p* = 0.04).

The cell viability (Table 4 A–D) was compromised by the addition of p-SWCNTs in all cell lines, which was started from 12 h of exposure at the lowest concentration (0.2 µg/mL). The increase in cell death and LDH release was time- and concentration-dependent and it differed slightly in various cell lines. The differences were due to the size of cells and rate of mitosis. The addition of low doses (0.2 µg/mL) of GO to cell cultures did not show cytotoxic activity in any cell line and 2 µg/mL of GO started to induce a moderate rate of cell death only in HEp-2 cells. The time of exposure has been shown to be an important factor for cell death and LDH release. The release of LDH and the percentage of cytotoxicity, which are reported in Table 5 A–D, increased slightly with longer exposure times to GO in the different cell lines but it never reached the same levels as those in the cells treated with p-SWCNTs. Figure 2 and Figure 3 show cytotoxicity in the cell lines after 24 h of treatment with p-SWCNTs and GO, respectively. In Figure 2, LDH release is inversely proportional to the cell viability and differences between controls (untreated cells), with 2 µg of p-SWCNTs for 24 h resulting in the highly significant release of LDH in all cell lines. Figure 3 shows that in the cells treated with GO for 24 h, the cell viability decreased significantly (*p* = 0.03) only in HEp-2 cells treated with 2 µg GO, while LDH release did not change significantly. The time- (up to 72 h) and concentration-dependent (5, 10 and 20 µg/mL) curves obtained in preliminary experiments with both nanomaterials showed that higher concentrations and longer times of exposure caused higher degrees of cell death and LDH release.

### 2.3. Morphological Changes in Cell Lines Treated with GO Nanosheets and p-SWCNTs

**Figure 4:**
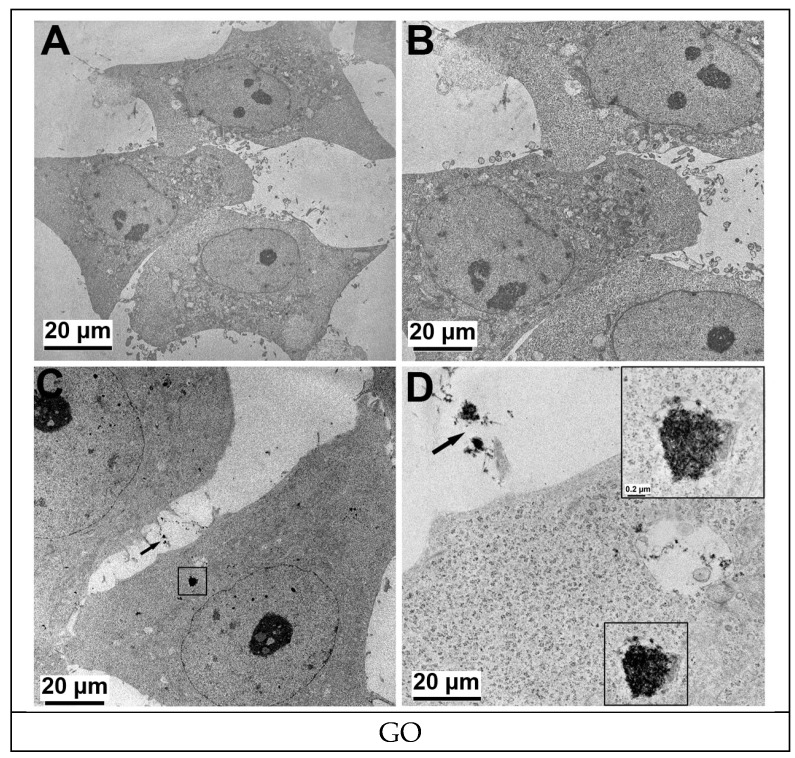
TEM images of GO-treated HeLa cells. (**A**,**B**) HeLa cells control (untreated) showing the normal nuclear and cellular morphology with no sign of sub-cellular compartment alteration. (**C**,**D**) HeLa cells treated with 2 µg/mL of GO for 24 h were able to incorporate GO (framed area, electron dense aggregate), which is also present in the extracellular space (arrow). Representative experiment.

**Figure 5:**
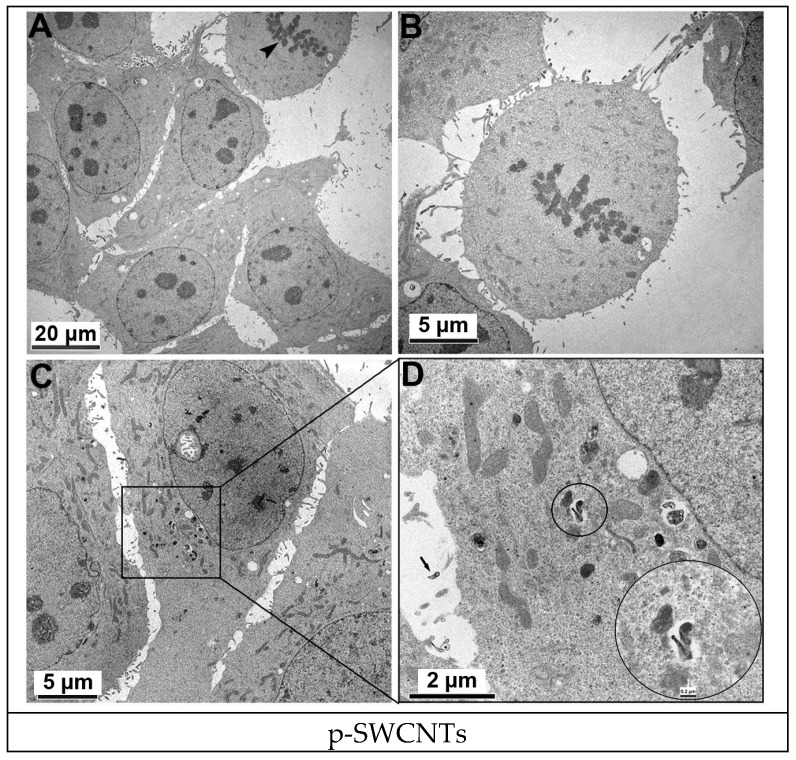
TEM images of p-SWCNTs-treated HeLa cells. (**A**,**B**) HeLa cells controls (untreated) during mitosis (arrowhead). (**C**,**D**) HeLa cells treated with 2 µg/mL of p-SWCNTs for 24 h were able to incorporate p-SWCNTs (circled area, electron dense tubular structure), which are also present in the extracellular space (arrow). Representative experiment.

The exposure of cells to 2 µg/mL of GO and 2 µg/mL of p-SWCNTs for 24 h led to a clearly visible internalization of these nanomaterials, which appeared to be similar to intracytoplasmic electron dense aggregates (GO) or tubular structures (p-SWCNTs) (Figure 4 and Figure 5, respectively). The vacuolization of cytoplasm was more evident when the cells were treated with p-SWCNTs than when treated with GO. In some cases, nanoparticles were found inside vacuoles (Figure 5).

**Figure 6:**
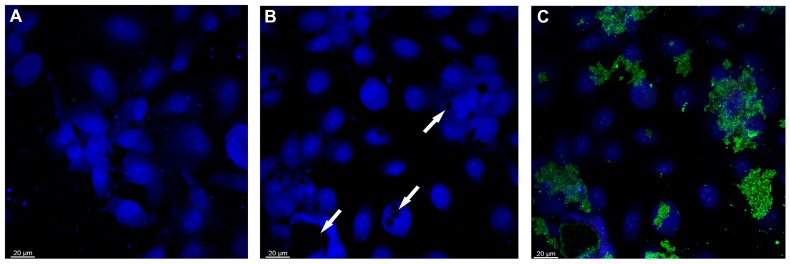
Confocal microscopy of (**A**) EaHy926 control cells, with nuclei stained in blue with DAPI (see Materials and Methods); (**B**) EaHy926 treated with 2 µg/mL of p-SWCNTs for 24 h (arrows on nuclear damage); and (**C**) p-SWCNTs in green. Representative experiment.

Confocal light scanning microscopy (CLSM) was used to study the morphological features seen in p-SWCNTs and GO treated-EaHy926 cells compared with control cells. Figure 6 shows the EaHy926 control cells (Figure 6A) and EaHy926 (Figure 6B) treated with 2 µg/mL of p-SWCNTs for 24 h. The cellular and nuclear damage (black spots) shown in Figure 6B were observed in the cells treated with p-SWCNTs at the lowest concentration. When the spectra signals are recorded in reflectance mode [27] (Figure 6C), the p-SWCNTs aggregates are seen as green spots in damaged nuclear areas. The switching from black into green spots in DAPI-stained nuclei depends on the accumulation and aggregation of p-SWCNTs and is a direct proof of damage, according to other authors [28].

**Figure 7:**
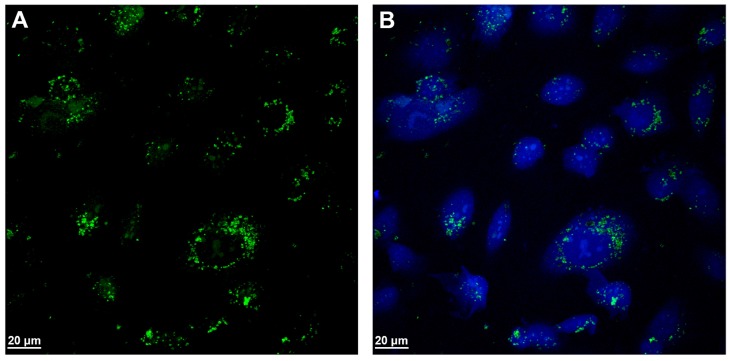
Confocal Laser Scanning Microscopy of EaHy926 treated with 2 µg/mL GO nanosheets; (**A**) GO in green and (**B**) nuclei stained in blue with DAPI and fluorescent GO in green. Representative experiment.

When GO was added to cell cultures under the same experimental conditions, the nuclei were not destroyed (Figure 7A,B and supplementary Figures S1–S3). The low green signal in cytoplasm is due to the GO nanosheets in the cytoplasm and no green signal is visible in the nuclei. All cell lines have been treated with the described amounts of both nanomaterials, while TEM or confocal microscopy have been performed in the samples from each cell line. The pictures shown in figures have been chosen as the examples of the most significant experiments.

## 3. Discussion

The present work demonstrated that the interactions of nanomaterials with human cell lines are affected by their size and chemical composition, while these interactions also depend on cell types. Regarding the composition of nanomaterials, the concern of researchers is that the substances that are used as precursors may be toxic for operators and for human cells when they are eventually administered as drug carriers.

The p-SWCNTs are obtained by chemical vapor deposition (CVD) using metals as catalysts, [29] while the PAHs (Polycyclic Aromatic Hydrocarbons) represent chemical residues that are adsorbed in the final SWCNT products [30]. Both compounds are a risk for workers because they produce airborne particulate matter. In order to monitor the actual risk related to the described inhaled substances in workplaces, various techniques may be applied [31].

In the present work, the p-SWCNTs were more cytotoxic than GO in the studied cell lines, which was demonstrated by the higher release of LDH and cell viability.

The GO nanosheets were obtained by the electrochemical exfoliation of solid Highly Oriented Pyrolytic Graphite (HOPG) rods, which resulted in highly purified GO. This newly synthesized GO does not contain or release metals or other substances in an aqueous solution, which was demonstrated by XRF spectroscopy and summarized in Table 1. When comparing the elemental analysis of the latter with that of p-SWCNTs, a substantial difference was found (Table 2). The presence of metal ions in nanomaterials is of concern as they may induce the production of ROS (Radical Oxygenated Species) in cells, may accumulate in macrophages and may contaminate the environment.

The oxygenated functionalities (i.e., the COOH groups) have been also detected by recording the negative z-potential values, according to the presence of the carboxylate anions, at physiological pH. In particular, the zeta potential value of −7.4 mV in the p-SWCNTs implies that it binds with strong cationic substances. In contrast, the lower value of −1.4 mV detected for GO suggests the π–π hydrophobic weak bonds are the most probable group that interacts with other chemical species present in the same solutions. These carboxylic groups could be used for further functionalization as they can be suited for binding various biological active molecules and for assembling new tools for drug delivery into cells. 

Confocal Laser Scanning Microscopy (CLSM) and TEM (Transmission Electron Microscopy) showed that the particles of both p-SWCNTs and GO nanosheets were located inside cells so the uptake of the nanomaterials used here was not a problem for single cells in our experimental conditions. In our previous work [32], we showed that GO nanoribbons had low toxicity in neuroblastoma cell lines, although we believe that the uptake of nanomaterials should be studied more extensively in tissues and in tridimensional models.

The p-SWCNTs and GO nanosheets were internalized within 24 h in different cell lines representing different tissues (HUVEC, EaHy926, HEp-2, HeLa). The variable values related to cytotoxicity shown in the different cell lines should be related to the different origin, growth rate and different activation of death modalities of cells. It has been reported [33] that the differentiated cells undergo necrosis when their membranes are damaged, while cancerous or fibroblast-like cells undergo apoptosis.

The evaluation of the plasma membrane integrity by trypan blue exclusion test and the release of LDH has shown high viability levels in all cell lines after treatment with GO nanosheets at 0.2 µg/mL. GO nanoribbons obtained from the chemical exfoliation of p-SWCNTs induced autophagy, which was caused by ROS induction in neuroblastoma cell lines. Instead, we have shown in this study that the GO nanosheets at low concentrations do not induce significant LDH release in various cell lines. The cytotoxic effects induced by p-SWCNTs were clear in all cell lines. In particular, apart from damaging cell membranes and inducing LDH release, the confocal microscopy showed nuclear damage. The shape of nanomaterials has been shown to play an important role in cytotoxicity as the nanotubes with their needle-like shape are similar to the asbestos fibers that can induce mesothelioma [34]. However, this concern has been adjusted as carbon nanotubes have not been shown to persist in organisms for as long as asbestos and they do not probably get to pleura by inhalation. Furthermore, it has not been demonstrated that the carbon nanotubes chronically activate macrophages and form granulomas. However, the size and shape of nanomaterials remain an important issue for biosafety. The GO nanosheets obtained in our laboratory are a promising nanomaterial to be functionalized and used as a drug carrier because they show low cytotoxicity in different cell lines and a homogeneous uptake in the reported cell lines. In conclusion, the results obtained here showed that GO synthesized by the water electrolytic oxidation of HOPG electrode might be considered a green synthesis approach as airborne particulate matter is not produced, while no strong oxidizing acids or other toxic chemicals are used [35]. However, more studies, including continuous monitoring in laboratories where these nanoproducts are produced and handled, are needed in order to reduce the health risks for workers handling nanoparticles and increase their biosafety.

## 4. Materials and Methods

### 4.1. Materials and Chemical Reagents

The p-SWCNTs were purchased from the Carbolex (Sigma Aldrich, Milan, Italy). GO was synthesized by the electrochemical exfoliation of HOPG (Highly Oriented Pyrolytic Graphite) working electrodes (HOPG rod electrodes were purchased from Good Fellow, TX, USA) according to the Patent (N° 102015000023739). The 2-(4-Amidinophenyl)-6-indolecarbamidine dihydrochloride (DAPI) was used for the nuclei staining in CLSM measurements, which was purchased from Sigma-Aldrich. All chemical reagents were purchased from Sigma Aldrich and used without further purification, unless otherwise specified. Ultra-pure water was produced by a Milli-Q system (Millipore, Billerica, MA, USA) and used for all chemical procedures.

### 4.2. Determination of Metal Content of Nanotube Samples

The metal quantification of the p-SWCNTs used in the present work was determined by using the ICP-OES apparatus (PE Sciex Elan 6000, Perkin Elmer, Norwalk, CT, USA). This quantitative measurement was carried out in 1 mL of the working medium supernatant, in which the p-SWCNTs were maintained in suspension for 10 days.

### 4.3. Air Oxidation of p-SWCNTs

Following one of the purification methodologies [36], 100 mg of carbon nanotubes was oxidized at 400 °C using an air flow of 12 mL/min (quartz tubular reactor of 14-mm diameter) for 1 h. To minimize the amount of metal oxide catalysts, the oxidized carbon nanotubes (by the physical air annealing procedures) were dispersed in 60 mL of 6 M HCl for 4 h, which involved using an ultrasonic bath. After this, they were filtered and washed until the pH of the solution was neutral. Finally, they were dried in the oven at 70 °C.

### 4.4. Synthesis and Characterization of GO Sheets

The GO sheets were obtained using electrochemical exfoliation/intercalation procedure from Highly Oriented Pyrolytic Graphite (HOPG rod electrodes, from Good Fellow, TX, USA), which was the solid precursor. The electrochemical exfoliation occurred in an electrolysis cell, where the Working Electrode (WE) was HOPG (φ = 3 mm) and the reference secondary electrode was a platinum rod (Pt; φ = 3 mm). These two electrodes were located at a distance of 5 cm in the electrolysis cell. A 0.1 M LiCl aqueous working medium was employed with a 12-V potential for 1 h. A step-by-step procedure for the electrochemical oxidative exfoliation of HOPG electrode is reported in the patent obtained in our laboratory in 2015 Patent (N° 102015000023739). The final GO product is used in liquid phase and directly collected from the electrolytic synthetic bath. An aliquot of 2 mL of the buffer with exfoliated GO (collected directly from the electrolysis cell) was diluted to 1:10,000 in PBS in order to obtain a final concentration of 1 mg/mL (as determined by using the conventional Faraday laws).

**Raman study.** The Raman spectra were collected both on a GO powder sample and on p-SWCNTs powder deposited on a Si (111) wafer at room temperature in the back-scattering geometry with a Renishaw spectrometer equipped with an air-cooled CCD detector and edge filters. A 488.0-nm emission line from an Ar^+^ ion laser was focused on the sample by a Leica DLML microscope using 5× or 20× objectives. After this, 10-s or 20-s accumulations were generally attained for each sample with an incident beam power of about 5 mW. The spectral resolution was 2 cm^−1^ and the spectra were calibrated using the 520.5 cm^−1^ line of a silicon wafer. The data analysis included the baseline removal and curve fitting using a Lorentzian function by Peakfit 4.12 software (Jandel, AISN Software, Erkrath, Germany).

**Infrared Spectroscopy (IR) study.** The IR spectra were recorded on a Perkin-Elmer Spectrum One FT-IR spectrometer. The samples were prepared in KBr pellets.

Z-potential measurements. The Z-potential measurements were obtained by using a Zetasizer Nano ZS equipment (Malvern, UK). This apparatus is equipped with a back-scattering detection mode (with an angle of 173°) and a He-Ne laser, which had a wavelength of 633 nm (Laser Doppler Velocimetry, Malvern, UK).

**Thermogravimetric Analysis (TGA**). The Thermal Analysis was performed by using a TGA/DSC1 Star System (Mettler Toledo) in N_2_ with a heating rate of 5 °C/min.

**Atomic Force Microscopy (AFM).** The AFM measurements were performed in air using a Veeco Multiprobe IIIa instrument (Veeco, Santa Barbara, CA, USA). The GO was deposited using an aqueous solution on the Mica substrates. The experiments were carried out at room temperature (20 °C) in tapping mode using Si tips with a spring constant of about 40 N/m and a typical curvature radius on the tip of 7 nm.

**X-ray Photoelectron Spectroscopy (XPS)*.*** The XPS measurements were carried out using an Omicron DAR 400 Al/Mg Kα non-monochromatic X-ray source and a VG-CLAM2 electron spectrometer. GO (1 mg/mL) was very-well dispersed in ethanol using an ultrasonic probe and then deposited on the silicon wafer.

### 4.5. Cell Lines

Different cell lines were used: Human Umbilical Vein Endothelial Cells (HUVEC) (ATCC, Manassas, VA, USA, CRL 1730); human cervix adenocarcinoma (HeLa) (DSMZ, ACC.57); EaHy926, an immortalized hybrid cell line derived from the fusion of HUVEC with lung carcinoma (A549) (ATCC, Manassas, VA, USA, CRL 2922); and human laryngeal epidermoid carcinoma (HEp-2), purchased from a Korean Cell Line Bank (KCLB No. 10023). HUVEC were cultured in EGM-2 BulletKit (CC-3162, Lonza, Basel, Switzerland). The other cell lines were cultured in Dulbecco's modified Eagle's media (DMEM), which was supplemented with 10% fetal bovine serum (FBS), 1% streptomycin-penicillin and 1% l-glutamine (Gibco, Thermo Fisher Scientific, Cambridge, MA, USA).

The cells were maintained as adherent cultures in Gibco tissue culture flasks under humidified atmosphere in 5% CO_2_ at 37 °C. The experiments were performed on cells that had grown up to 60–70% confluency.

The GO and p-SWCNTs suspensions were dispersed in distilled H_2_O at a concentration of 1 mg/mL and sonicated for 2 h in a 4.25 L bath type ultrasonic unit (Bransonic 220, Branson, MO, USA). Following sonication, the suspensions were centrifuged at 13,000 rpm for 30 min and the supernatants were transferred to fresh tubes. Both p-SWCNTs and GO were sonicated for another hour, before a single amount was very well-dispersed in the tissue culture medium (at the desired concentration). This single amount was ready to be used for cell treatment experiments.

Transmission electron microscopy (TEM). Cells (treated or not with GO or p-SWCNTS) were fixed in 1% OsO_4_ for 2 h and progressively dehydrated by ethanol/water to an absolute ethanol series. They were treated with propylene oxide before being embedded in EPON 812. Ultrathin sections were counterstained with uranyl acetate and lead citrate before being studied with a Hitachi 7100 transmission electron microscope (Hitachi, Tokyo, Japan).

Confocal Laser Scanning Microscopy (CLSM). A Laser Scanning Confocal Microscope (Olympus FV1000, Olympus, Hamburg, Germany) was used for the multi-channel imaging studies. The optical glass slides (0.17 mm of thickness), suitable for the CLSM measurements, were sterilized and placed in the wells of a cell culture plate, before the cells were seeded at a concentration of 10^5^/mL. The p-SWCNTs and GO dispersions were added to the culture medium at the concentrations and times indicated in the Results section. At the end of incubation time, the cells were washed with PBS and fixed with 4% paraformaldehyde for 20 min. After washes, cells were treated with 0.1 M glycine in PBS for 10 min and then permeabilized with 0.1% Triton in PBS solution for 5 min. After washing, the nuclei were stained with DAPI for 5 min and then mounted with a cover slip and a drop of mounting solution (glycerol:PBS, 3:2). The readings were carried out with a confocal microscope (Zeiss LSM-510, Oberckoken, Germany). The laser sources were a diode at 405 nm for the blue channel and Argon at 488 nm for the green channel. The green signal from the p-SWCNTs and GO aggregates (488 nm for excitation and 510 nm for emission) was recorded in the cells treated with nanoparticles at different times of incubation [37].

Trypan blue assay. The cell number and cell viability were determined by using the Trypan blue exclusion test. Cells in the exponential phase of growth (1 × 10^5^/mL) were seeded into a 6-well microplate and incubated for 6, 12, 24 and 48 h with 0.2 µg/mL or 2 µg/mL of p-SWCNTs and GO, respectively. The untreated cells were used as negative controls. The p-SWCNTs and GO stock solutions (1 mg/mL in PBS) were sonicated for 2–4 h (4 °C), before being diluted in sterile culture medium and added to the cell cultures. At the end of incubation time, cells were mechanically scraped off, resuspended in fresh medium and incubated for 30 min with gentle shaking at 37 °C in an atmosphere of 5% CO_2_. An aliquot was then diluted (1:1) with a solution of 0.4% Trypan blue stain. After a few minutes at room temperature, cells were counted under an optical microscope in a Thoma hemocytometer chamber by two different operators. The values are expressed as % of viable cells. The cell viability in control samples was always 97–98%.

### 4.6. LDH Assay

The cytotoxic effects of 0.2 and 2 µg/mL suspensions of p-SWCNTs and GO were measured in the treated EaHy926, HEp-2, HUVEC and HeLa supernatants by quantitating the release of lactate dehydrogenase (LDH) at different treatment times (6, 12, 24 and 48 h). Following incubation, an aliquot (200 µL/well) of treated cells or untreated cells was centrifuged for 5 min at 4 °C and 13,000 rpm. Supernatants were collected for the LDH measurements according to the manufacturer’s instructions (Thermo scientific, Cambridge, MA, USA). The assay was performed in a 96-well plate, while the absorbance was measured at 490 and 680 nm by using a UV-Vis spectrophotometer (Jenway, Genova, Italy). LDH activity was obtained by subtracting the 680-nm value from the 490-nm absorbance. The preliminary experiments were set in each cell line to determine the maximum LDH activity that was necessary for the determination of the percentage of cytotoxicity according to this formula:

(1) $$\%\;\mathrm{cytotoxicity}=\;\frac{\mathrm{Treated}\;\mathrm{cells}\;\mathrm{LDH}\;-\;\mathrm{Control}\;\mathrm{cells}\;\mathrm{LDH}}{\mathrm{Maximum}\;\mathrm{LDH}\;-\;\mathrm{Control}\;\mathrm{cells}\;\mathrm{LDH}\;}\;\times \;100$$

The maximum LDH activity was 1.2 (A 490–680 nm) in each cell line.

### 4.7. Statistics

All statistical analyses were carried out using Kaleida Graph version 4.5.1 (Synergy Software Inc., Reading, PA, USA). The data are expressed as means ± standard deviation. The differences between the differently treated cell populations were analyzed using Student’s *t* test. *p* < 0.05 was considered to indicate a statistically significant difference. The significant *p* values are reported in figure legends.

## Figures and Tables

**Table 1:** Physico-chemical properties of GO.

**Chemical Properties**	**Metallic Elements**
Elemental analysis ^a^ (% *w/w*)	Si (n.d) S (n.d) Ca (n.d.) Cr (n.d.) Fe (n.d.) Co (n.d.)
**Physical Properties**	**Range Values**
Surface Area (µm^2^) ^b^	0.1–3.0
Thickness (nm) ^b^	1.2 ± 0.3
Number of Layer ^b^	2 (bilayer)
Weight loss % (TGA) ^c^	0.42 ± 0.40
Acidic sites (nmol/mg) ^d^	4.02 ± 0.23
Extent of defects (I_D_/I_G_) ^e^	0.10

^a^ Evaluated by XRF spectroscopy (mean ± SE). ^b^ Evaluated by AFM analysis, where the average size of GO sheets was obtained by measuring a significant number of samples (average was calculated using 80 sheets per GO). ^c^ Mean ± SD. ^d^ Acidic sites evaluated as described in reference [25] (mean ± SD). ^e^ Evaluated by Raman spectroscopy. n.d.: not detectable.

**Table 2:** Binding Energies (BE/eV) and deconvoluted peaks (%) for C1s of GO.

Peak BE (eV)	C1s At. %	Functional Groups
284.4	59.0	C-C
285.4	16.0	C-OH
286.6	14.0	C-O
287.7	7.0	C=O
289.0	4.0	C(O)O
290.7	2.2	π–π *

π * refers to antibonding π molecular orbitals, with the highest energy level.

**Table 3:** Physico-chemical properties of p-SWCNTs.

**Chemical Properties**	**Metallic Elements**
Elemental analysis ^a^ (% *w/w*)	Si 0.20 ± 0.06 S 0.47 ± 0.04 Ca 1.14 ± 0.31 Cr 1.03 ± 0.15 Fe 1.00 ± 0.25 Co 3.57 ± 0.56
**Physical Properties**	**Range Values**
Diameter (nm) ^b^	2.35 ± 0.40
Length (µm) ^b^	0.82 ± 0.42
Weight loss % (TGA) ^c^	0.50 ±0.50
Acidic sites (nmol/mg) ^d^	24.25 ± 1.10
Extent of defects (I_D_/I_G_) ^e^	0.94

^a^ Evaluated by XRF spectroscopy (mean ± SE). ^b^ Evaluated by TEM analysis (mean ± SD). ^c^ Mean ± SD. ^d^ Acidic sites evaluated as described in reference [26] (mean ± SD). ^e^ Evaluated by Raman spectroscopy.

**Table 4:** Viability test for different type of cells.

A. EaHy926					
Time (h)	CTRL	p-SWCNTs 0.2 μg/mL	p-SWCNTs 2 μg/mL	GO 0.2 μg/mL	GO 2 μg/mL
6	99.40	94.40	84.32	95.50	95.20
12	98.20	80.20	78.20	92.60	91.60
24	98.20	70.20	62.50	92.60	89.40
48	98.20	56.60	47.30	92.00	88.60
B. HEp-2					
Time (h)	CTRL	p-SWCNTs 0.2 μg/mL	p-SWCNTs 2 μg/mL	GO 0.2 μg/mL	GO 2 μg/mL
6	98.60	90.30	77.30	94.50	93.80
12	97.80	80.00	62.20	89.80	86.00
24	96.00	69.00	58.00	87.00	82.00
48	91.20	64.20	45.30	81.40	80.00
C. HUVEC					
Time (h)	CTRL	p-SWCNTs 0.2 μg/mL	p-SWCNTs 2 μg/mL	GO 0.2 μg/mL	GO 2 μg/mL
6	98.30	92.40	82.40	98.00	97.50
12	97.60	85.60	70.70	97.80	96.20
24	98.20	72.20	62.80	96.70	95.70
48	97.30	66.00	58.20	96.50	94.20
D. HeLa					
Time (h)	CTRL	p-SWCNTs 0.2 μg/mL	p-SWCNTs 2 μg/mL	GO 0.2 μg/mL	GO 2 μg/mL
6	97.20	85.70	84.60	96.30	95.20
12	98.50	75.30	73.90	96.60	94.20
24	97.60	72.20	65.00	95.40	91.60
48	96.70	67.00	65.00	93.20	89.20

% of viable cells determined by Trypan blue exclusion test, as described in Materials and Methods. The values represent the mean obtained in 3 experiments. SD were between ±1% and ±2%.

**Table 5:** LDH activity and % of cytotoxicity.

A. EaHy926					
Time (h)	CTRL	p-SWCNTs 0.2 μg/mL	p-SWCNTs 2 μg/mL	GO 0.2 μg/mL	GO 2 μg/mL
12	0.16	0.28 (12%)	0.32 (15.4%)	0.18 (1.9%)	0.23 (6.7%)
24	0.18	0.42 (23.5%)	0.52 (33.3%)	0.22 (3.9%)	0.28 (9.8%)
48	0.25	0.54 (30.5%)	0.66 (43%)	0.36 (11.6%)	0.40 (15.8%)
B. HEp-2					
Time (h)	CTRL	p-SWCNTs 0.2 μg/mL	p-SWCNTs 2 μg/mL	GO 0.2 μg/mL	GO 2 μg/mL
12	0.18	0.38 (16.6%)	0.42 (23.5%)	0.19 (1.0%)	0.25 (6.8%)
24	0.21	0.41 (20.2%)	0.50 (29.3%)	0.24 (2.9%)	0.27 (6%)
48	0.26	0.46 (21.3%)	1.22 (102%)	0.29(3.2%)	0.35 (9%)
C. HeLa					
Time (h)	CTRL	p-SWCNTs 0.2 μg/mL	p-SWCNTs 2 μg/mL	GO 0.2 μg/mL	GO 2 μg/mL
12	0.14	0.18 (1.9%)	0.30 (15%)	0.14 (0%)	0.15 (0.94%)
24	0.15	0.28 (13.2%)	0.31 (16%)	0.16 (0.9%)	0.22 (6.6%)
48	0.16	0.49 (31.7%)	0.59 (41.3%)	0.18 (1.9%)	0.28 (11.5%)
D. HUVEC					
Time (h)	CTRL	p-SWCNTs 0.2 μg/mL	p-SWCNTs 2 μg/mL	GO 0.2 μg/mL	GO 2 μg/mL
12	0.12	0.31 (17.6%)	0.45 (30.6%)	0.13 (0.9%)	0.22 (9.3%)
24	0.14	0.34 (18.9%)	0.50 (33.9%)	0,.15 (0.9%)	0.26 (11.3%)
48	0.15	0.43 (26.6%)	0.80 (61.9%)	0.17 (1.9%)	0.31 (15.4%)

LDH activity was determined in the different cell lines, as described in Materials and Methods. LDH values are expressed as the difference between readings at 490 and 680 nm (A 490–680 nm). The % of cytotoxicity shown after each treatment (%) was determined by applying the formula reported in Materials and Methods. The determinations were performed 3 times and each reported value represents the mean. The SDs were between ±0.09 and ±0.2.

## Supplementary Materials

Supplementary materials can be found at http://www.mdpi.com/1422-0067/19/5/1316/s1.
