# Supplementary material for: Metal Free Graphene Oxide (GO) Nanosheets and Pristine-Single Wall Carbon Nanotubes (p-SWCNTs) Biocompatibility Investigation: A Comparative Study in Different Human Cell Lines
Source: Int J Mol Sci. 2018 Apr 28;19(5):1316. doi: 10.3390/ijms19051316 (PMC5983736; doi:10.3390/ijms19051316)
Supplement: Supplementary file 1 [file ijms-19-01316-s001.pdf]

Metal free Graphene Oxide (GO) nanosheets and pristine-Single Wall Carbon Nanotubes (p-SWCNTs) biocompatibility investigation: a comparative study in different human cell lines.

F. Valentini[1,2°*], E. Mari[3°], A. Zicari[3], A. Calcaterra[2], M. Talamo[2], M. G. Scioli[4], A. Orlandi[4], and S. Mardente[3]

1. Department of Sciences and Chemical Technologies, University of Rome Tor Vergata, Via della Ricerca Scientifica 1, 00133 Rome (Italy)
2. INUIT Foundation, University of Rome Tor Vergata, via dell'Archigginasio, Rome (Italy)
3. Department of Experimental Medicine, University of Rome Sapienza, Viale Regina Elena 324, 00161 Rome (Italy)
4. Department of Medicine, Pathological Anatomy, University of Rome Tor Vergata, Viale Oxford 81, 00133 Rome (Italy)

Supplementary Material

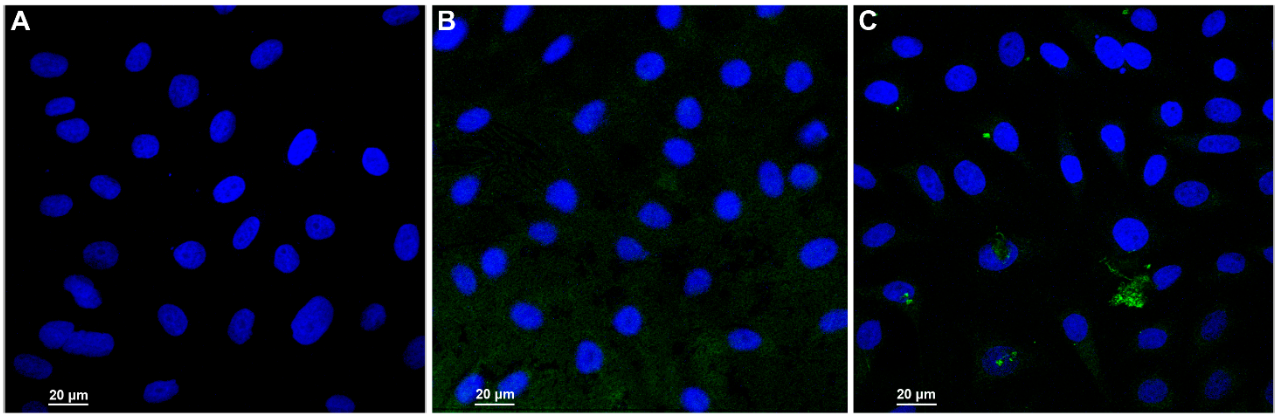

Figure S1: Confocal microscopy of **A**: HeLa control cells, nuclei are stained in blue with DAPI, **B**: HeLa treated with 2 μg/mL of GO for 24 h (GO in green) and **C**: HeLa treated with 2 μg/mL of p-SWCNTs for 24 h (p-SWCNTs in green). Representative experiment.

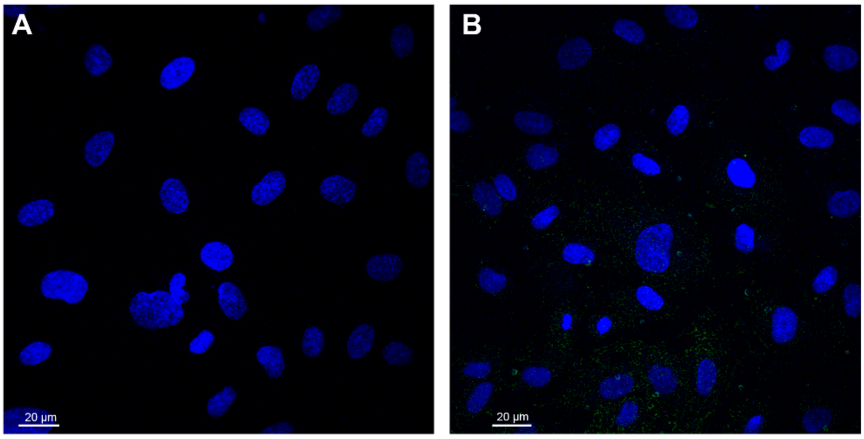

Figure S2: Confocal microscopy of **A**: HUVEC control cells, nuclei are stained in blue with DAPI and **B**: HUVEC treated with 2 µg/mL of GO for 24 h (GO in green). Representative experiment.

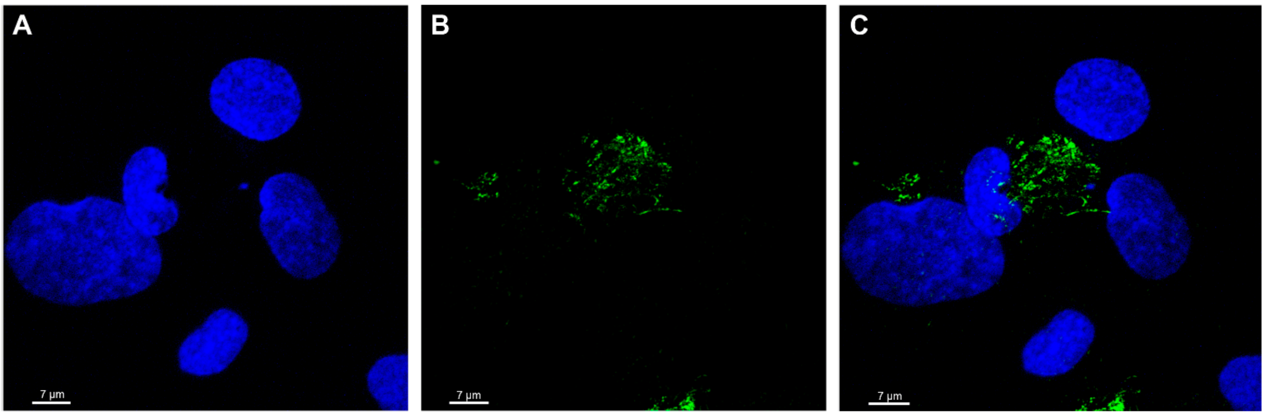

Figure S3: Confocal microscopy of **A**: HUVEC cells treated with 2 µg/mL of p-SWCNTs for 24 h, nuclei stained with DAPI, **B**: p-SWCNTs and **C**: merge of blue nuclei and green SWNCTs. Representative experiment.
